# Distinct volumetric features of cerebrospinal fluid distribution in idiopathic normal-pressure hydrocephalus and Alzheimer’s disease

**DOI:** 10.1186/s12987-022-00362-8

**Published:** 2022-09-01

**Authors:** Jaehwan Han, Myoung Nam Kim, Ho-Won Lee, Shin Young Jeong, Sang-Woo Lee, Uicheul Yoon, Kyunghun Kang

**Affiliations:** 1 Department of Biomedical Engineering, School of Medicine, Kyungpook National University, Daegu, South Korea; 2 Department of Neurology, School of Medicine, Kyungpook National University, 680 Gukchaebosang-ro, Jung-gu, Daegu, 41944 South Korea; 3 Brain Science and Engineering Institute, Kyungpook National University, Daegu, South Korea; 4 Department of Nuclear Medicine, School of Medicine, Kyungpook National University, Daegu, South Korea; 5 Department of Biomedical Engineering, Daegu Catholic University, 13-13 Hayang- ro, Hayang-eup, Gyeongsan, Gyeongbuk 38430 South Korea

**Keywords:** Idiopathic normal-pressure hydrocephalus, Cerebrospinal fluid space, Magnetic resonance imaging, Alzheimer’s disease

## Abstract

**Objective:**

The aims of the study were to measure the cerebrospinal fluid (CSF) volumes in the lateral ventricle, high-convexity subarachnoid space, and Sylvian fissure region in patients with idiopathic normal-pressure hydrocephalus (INPH) and Alzheimer’s disease (AD), and to evaluate differences in these volumes between INPH and AD groups and healthy controls.

**Methods:**

Forty-nine INPH patients, 59 AD patients, and 26 healthy controls were imaged with automated three-dimensional volumetric MRI.

**Results:**

INPH patients had larger lateral ventricles and CSF spaces of the Sylvian fissure region and smaller high-convexity subarachnoid spaces than other groups, and AD patients had larger lateral ventricles and CSF spaces of the Sylvian fissure region than the control group. The INPH group showed a negative correlation between lateral ventricle and high-convexity subarachnoid space volumes, while the AD group showed a positive correlation between lateral ventricle volume and volume for CSF spaces of the Sylvian fissure region. The ratio of lateral ventricle to high-convexity subarachnoid space volumes yielded an area under the curve of 0.990, differentiating INPH from AD.

**Conclusions:**

Associations between CSF volumes suggest that there might be different mechanisms between INPH and AD to explain their respective lateral ventricular dilations. The ratio of lateral ventricle to high-convexity subarachnoid space volumes distinguishes INPH from AD with good diagnostic sensitivity and specificity. We propose to refer to this ratio as the VOSS (ventricle over subarachnoid space) index.

## Introduction

Idiopathic normal-pressure hydrocephalus (INPH) is a treatable neurologic disorder characterized by ventricular dilatation (Evans’ index > 0.3), normal cerebrospinal fluid (CSF) pressure at lumbar puncture, and a symptom triad of cognitive impairment, gait disturbance, and urinary dysfunction [1]. Ventriculomegaly is the distinctive morphologic characteristic of INPH.

Alzheimer’s disease (AD) is the most frequent cause of dementia in the elderly. While ventriculomegaly is the primary morphologic characteristic of INPH, it is also seen in AD [2]. AD patients show diffuse cerebral atrophy, which leads to secondary ventricular enlargement. Further, cerebral atrophy as a consequence of aging can extend ventricular dilatation. In clinical situations, gait disturbance additionally is well known to be frequent in AD [3].

INPH with non-obstructive ventricle enlargement can be difficult to differentiate from AD with ex vacuo ventricular enlargement based on standard MRI findings alone [1]. And the diagnosis of INPH is complicated by the variability that exists in its clinical presentation and course [1]. Nevertheless, the diagnosis of INPH is also important because INPH is regarded as a reversible neurodegenerative disease. As a result, additional tests for differential diagnosis are often necessary.

Tightness in high-convexity and medial subarachnoid spaces, which is most noticeable on coronal MRI sections, is a well-known feature of INPH [4]. As a consequence, high-convexity tightness has been suggested as an alternative imaging feature to discriminate INPH from AD [2]. The imaging characteristics of ventriculomegaly, high-convexity tightness, and Sylvian fissure dilation has been called “disproportionately enlarged subarachnoid space hydrocephalus” (DESH) [2]. The usefulness of DESH in distinguishing INPH from other neurologic diseases has been noted in several studies [2]. However, evaluating DESH on MRI images can be difficult for less-experienced physicians. The qualitative nature of this visual interpretation has been suggested to be the major drawback for its use clinically [5]. In general, automated volumetric measurements are more objective and reliable and can provide exact measurements of regional brain volume in comparison with visual interpretations [6].

There have been three studies about volumetric measurements of CSF spaces, including the lateral ventricles, high-convexity subarachnoid space, and CSF spaces of the Sylvian fissure region in INPH patients; however, these studies only included 11, 12, or 19 INPH participants, respectively [7–9]. One of these studies used a manual delineation method for volumetric analysis [7]. However, manual tracing of ROI on successive brain slices is time-consuming and can include substantial errors during measurement [8]. Two of these reported that voxel-based morphometry (VBM)-based CSF space analysis can be used to determine the characteristic alteration of the CSF space in INPH patients [8, 9]. However, they examined only two CSF space volumes, as they combined the lateral ventricle and Sylvian fissure area in one volume and the high-convexity subarachnoid space in another. And a previous study with 29 INPH patients reported that the ratio between the two-dimensional areas of the Sylvian fissure and the subarachnoid space at the vertex was a reliable tool to easily quantify DESH on computed tomography scans of patients with suspected INPH, but they did not examine AD patients [5].

In this study, we investigated the volumes of key CSF spaces (i.e., the lateral ventricles, high-convexity subarachnoid space, and CSF spaces of the Sylvian fissure region) utilizing an automated three-dimensional volumetric approach in 3 groups: (1) INPH patients with a positive response to a CSF tap test (CSFTT), (2) age-matched AD patients, and (3) healthy controls. The aims of the study were to evaluate differences in CSF space volumes among the 3 groups and to investigate relationships between lateral ventricle volume, volume for high-convexity subarachnoid space, and volume for CSF spaces of the Sylvian fissure region in INPH and AD patients. Since dilated lateral ventricles and high-convexity tightness often occur together in INPH, we investigated a ratio of the two for distinguishing INPH from AD.

## Methods

### Participants

INPH patients who visited the Center for Neurodegenerative Diseases of Kyungpook National University Chilgok Hospital, South Korea, from June 2013 to March 2017 were prospectively recruited. The INPH diagnosis was made using Relkin et al. criteria [1]. A lumbar tap removing 30–50 ml of CSF was done on each INPH patient. After the CSF tap, patients were re-evaluated with the Korean-Mini Mental State Examination (K-MMSE), the INPH Grading Scale (INPHGS), and the Timed Up and Go Test (TUG). Gait changes were evaluated multiple times over 7 days following the tap, and changes in cognition and urination were evaluated at 1 week. CSFTT response was defined using these 3 major scales [10]. INPH patients who had a positive response to the CSFTT according to the Japanese guidelines for INPH were enrolled [10].

AD patients and healthy controls were chosen randomly from our hospital and were matched to INPH patients according to age. AD was diagnosed using McKhann et al. criteria [11]. The criteria for healthy controls were as follows: normal neurological status on examination; no active neurological, systemic, or psychiatric disorders; and the ability to function independently. Global cognition of healthy controls was also assessed using the K-MMSE.

### MRI imaging acquisition

MRI data were obtained using a 3.0 T system (GE Discovery MR750, GE Healthcare). Three-dimensional T1-weighted, sagittal, and inversion-recovery fast spoiled gradient echo (IR-FSPGR) MRI images of the whole head, designed to optimally discriminate between brain tissues (sagittal slice thickness 1.0 mm, no gap, TR = 8.2 ms, TE = 3.2 ms, flip angle 12°, matrix size 256 × 256 pixels, and field of view = 240 mm), were acquired.

### Image analysis

In order to define an unbalanced distribution of the CSF spaces, the following image preprocessing steps were applied. At first, the MR images in native space were registered into the International Consortium for Brain Mapping (ICBM) 152 symmetric template using a linear transformation and corrected for intensity non-uniformity artifacts [12, 13]. Secondly, we removed all unnecessary parts of the MR image including the skull, extracranial tissue, cerebellum, and brainstem by an automated brain extraction algorithm [14], and an artificial neural network classifier was applied to identify cerebral tissues into gray matter (GM), white matter (WM), and CSF [15]. Partial volume levels, MRI intensity-mixing at the tissue interfaces due to the finite resolution of the imaging device, were estimated and corrected using a trimmed minimum covariance determinant method [16].

We adopted the graph cuts algorithm combined with an atlas-based segmentation to define the lateral ventricle, which would use a priori information for the following graph cuts algorithm [17]. A manually drawn lateral ventricle on the ICBM 152 symmetric template was used to define the lateral ventricle from individual data using nonlinear registration [17]. It provided a priori information for the graph cuts algorithm as foreground seeds, which was used to find a global minimum of energy function with minimum cut/maximum flow algorithms on the graph [17]. The definition of initial seeds was further elaborated by incorporating an estimation of partial volume probabilities at each voxel [17]. After graph cut computation, the segmentation result was finely post-processed by a morphological opening to reduce over- and under-segmented parts [18].

The Talairach coordinate system has a proportional grid based on stereotaxic spaces and consists of the tessellation of the brain into sectors of 1056 cells [19]. These cells enable defining particular regions or anatomical structures for selecting homologous cells and be useful for inter-subject comparisons. Before dividing into the proportional grid, all data were aligned to a stereotaxic space to be set the anterior commissure (AC) and the posterior commissure (PC) that lay on a straight horizontal line, the so-called AC-PC line [20]. We overlaid the Talairach grid onto each brain image in stereotaxic space. Previously described approaches were used to define regions of interest (ROIs) in the high-convexity area and in the Sylvian fissure region [21]. Briefly, the extreme top, bottom, left, right, anterior, and posterior edges of the eroded image were determined, and they defined the edges of the Talairach bounding box [21]. In the anterior to posterior direction, four equal divisions were created anterior to the AC and posterior to the PC [21]. The region between the AC and PC was divided into three equal sections [21]. From superior to inferior, the brain was divided into eight equal divisions above the AC-PC, and into four equal divisions below the AC-PC [21]. Each of the left and right hemispheres was divided into four equal sagittal regions [21]. Coordinates defined below were expressed relative to the Talairach reference planes as the number of grid coordinates [e.g., vPC(− 2) = two vertical grid lines posterior to the PC] [21].

#### High-convexity area

The inferior-superior extent of this region is from AC-PC(+ 3) to AC-PC(+ 8). This region extends sagittally from the midline (M) to M(± 1 sagittal grid divisions). The anterior-posterior extent of this region is from vAC(+ 1) to vPC(0).

#### Sylvian fissure region

The inferior-superior extent of this region is from AC-PC(− 2) to AC-PC(+ 3). The medial border is defined by M(± 2). The lateral border is defined by M(± 4). The anterior-posterior extent of this region is from vAC(+ 2) to vPC(− 1).

**Fig. 1:**
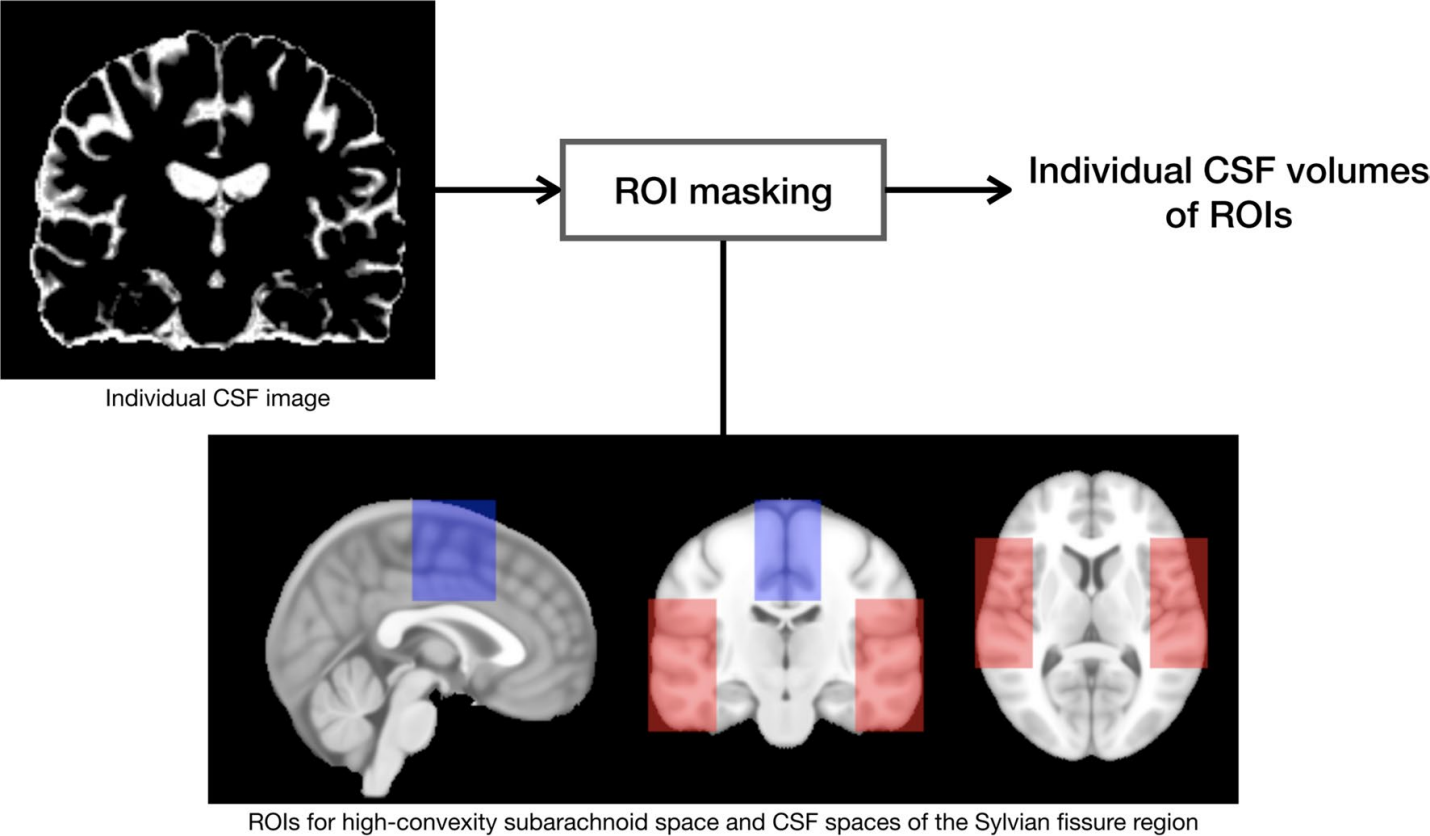
The processes to investigate volume of CSF spaces, including the high-convexity subarachnoid space and CSF spaces of the Sylvian fissure region. Individual CSF images were segmented from 3D-T1-weighted images. Two regions of interest (ROIs) were created for the high-convexity area (blue) and Sylvian fissure region (red), and these ROIs defined by Talairach grid divisions were shown in the ICBM 152 stereotaxic space

The amount of CSF in the high-convexity area and Sylvian fissure region was estimated from the CSF-classified map limited by each cell (Fig. 1). In addition to this procedure, the largest connected component labeling operation was applied to the estimation of CSF in the Sylvian fissure region in order to remove false positives [17]. For volumetric measurement, extracted CSF images were inversely registered into each native space. And then, normalization of the regional CSF volumes by intracranial volume (ICV) was applied to compensate for inter-individual variability in brain morphology and total head size, which was obtained by an automated brain extraction algorithm in the preprocessing step. Normalized CSF space volumes were expressed as ratios of ICV.

### Statistical analysis

R version 4.0.3 (https://www.r-project.org) was used for statistical analysis. The CSF space volumes of the INPH, AD, and control groups were compared by analysis of variance or Kruskal–Wallis tests, followed by Tukey’s post-hoc analysis. Pearson’s correlations were employed to investigate relationships between lateral ventricle volume and volume for high-convexity subarachnoid space and volume for CSF spaces of the Sylvian fissure region in patients with INPH and AD. Diagnostic accuracy of the lateral ventricle volume/high-convexity subarachnoid space volume ratio in distinguishing INPH from AD was calculated using area under the curve, sensitivity, specificity, and cutoff levels obtained using receiver operating characteristic (ROC) curves. Statistical significance was set at *P* < 0.05.

## Results

We enrolled 49 patients with INPH, 59 patients with AD, and 26 healthy controls. Patients and controls are characterized in Table 1. There were no significant age differences between the three groups.

**Table 1:** Characterization of patients and controls at baseline

Characteristics	Controls (n = 26)	INPH (n = 49)	AD (n = 59)
Age, year, mean ± SD	71.7 ± 4.1	73.5 ± 5.4	71.7 ± 8.1
Male gender, number (%)	10 (38.5)	30 (61.2)	14 (23.7)
Education, year, median (IQR)	12.0 (6.0–16.0)	9.0 (6.0–12.0)	6.0 (6.0–12.0)
Duration of symptoms, year, median (IQR)		2.0 (1.0–4.0)	2.5 (1.0–3.0)
K-MMSE, median (IQR)	27.0 (26.0–29.0)	21.0 (17.0–25.0)	19.0 (15.0–22.0)

Data are presented as mean ± SD for normally distributed variables and median (25–75th percentile, IQR) for non-normally distributed variables

*INPH* idiopathic normal-pressure hydrocephalus, *AD *Alzheimer’s disease, *SD *standard deviation, *IQR *interquartile range, *K-MMSE *Korean version of Mini-Mental State Examination

**Fig. 2:**
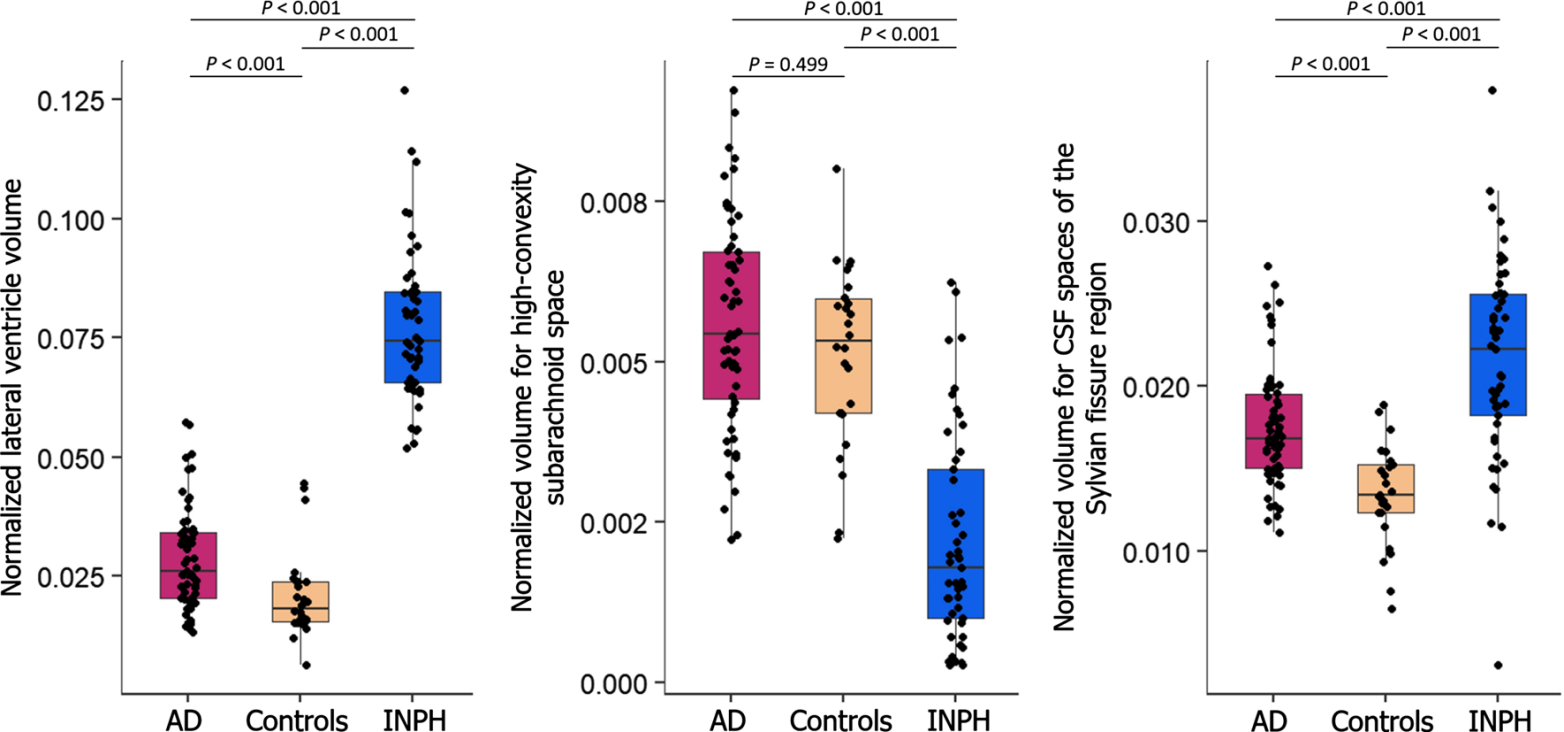
Box and whisker plots of the normalized volumes for lateral ventricles, high-convexity subarachnoid space, and CSF spaces of the Sylvian fissure region for three groups. Normalized CSF space volumes were expressed as regional volume/intracranial volume

The results of volume measurement are summarized in Table 2. Box plots with individual data points for all subjects in each of the three clinical groups are illustrated in Fig. 2 for the normalized volumes for lateral ventricles, high-convexity subarachnoid space, and CSF spaces of the Sylvian fissure region. The mean volumes of lateral ventricles and CSF spaces of the Sylvian fissure region in the INPH group were significantly larger than in the other two groups. The mean volumes of lateral ventricles and CSF spaces of the Sylvian fissure region in the AD group were also significantly larger than in the control group. The mean volume of high-convexity subarachnoid space in the INPH group was significantly smaller than in the other two groups. There were no significant differences in high-convexity subarachnoid space volume between the AD and control groups.

**Table 2:** Results of measurement of the normalized CSF space volume

Region	Controls	INPH	AD	Statistical comparison		
	(n = 26)	(n = 49)	(n = 59)	INPH versus controls	AD versus controls	INPH versus AD
Lateral ventricle^a^	0.018 (0.015–0.024)	0.074 (0.066–0.085)	0.026 (0.020–0.034)	*P* < 0.001	*P* < 0.001	*P* < 0.001
High-convexity subarachnoid space^a^	0.005 (0.004–0.006)	0.002 (0.001–0.003)	0.005 (0.004–0.007)	*P* < 0.001	*P* = 0.499	*P* < 0.001
CSF spaces of the Sylvian fissure region^a^	0.013 (0.012–0.015)	0.022 (0.018–0.026)	0.017 (0.015–0.020)	*P* < 0.001	*P* < 0.001	*P* < 0.001

Data are presented as median (25–75th percentile, IQR) for non-normally distributed variables

Normalized volume = (regional volume)/(intracranial volume)

^a^Significant intergroup difference with Kruskal–Wallis analysis (*P* < 0.001)

*INPH* idiopathic normal-pressure hydrocephalus, *AD *Alzheimer’s disease, *IQR *interquartile range

**Fig. 3:**
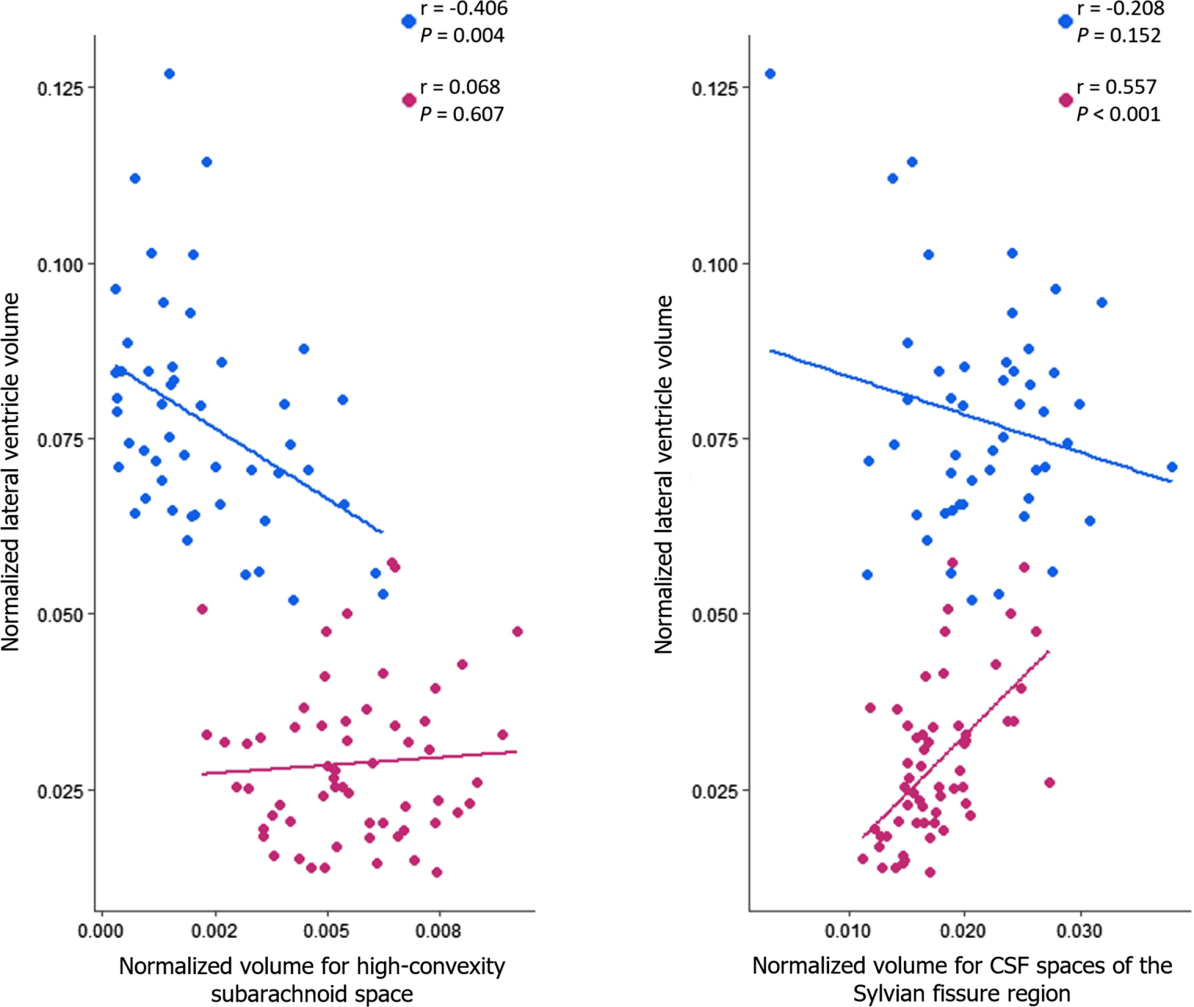
Relationship between normalized lateral ventricle volume and normalized volume for high-convexity subarachnoid space and normalized volume for CSF spaces of the Sylvian fissure region in patients with INPH and AD. Normalized CSF space volumes were expressed as regional volume/intracranial volume. Blue dots represent INPH subjects while red dots represent AD subjects

The INPH group showed a significant negative correlation between normalized lateral ventricle volume and normalized volume for high-convexity subarachnoid space (r = − 0.406, *P* = 0.004), whereas no correlation between these two measures was found in the AD group (r  = 0.068, *P*  = 0.607) (Fig. 3). The AD group showed a significant positive correlation between normalized lateral ventricle volume and normalized volume for CSF spaces of the Sylvian fissure region (r = 0.557, *P* < 0.001), whereas no correlation between these two measures was found in the INPH group (r = − 0.208, *P* = 0.152) (Fig. 3).

**Fig. 4:**
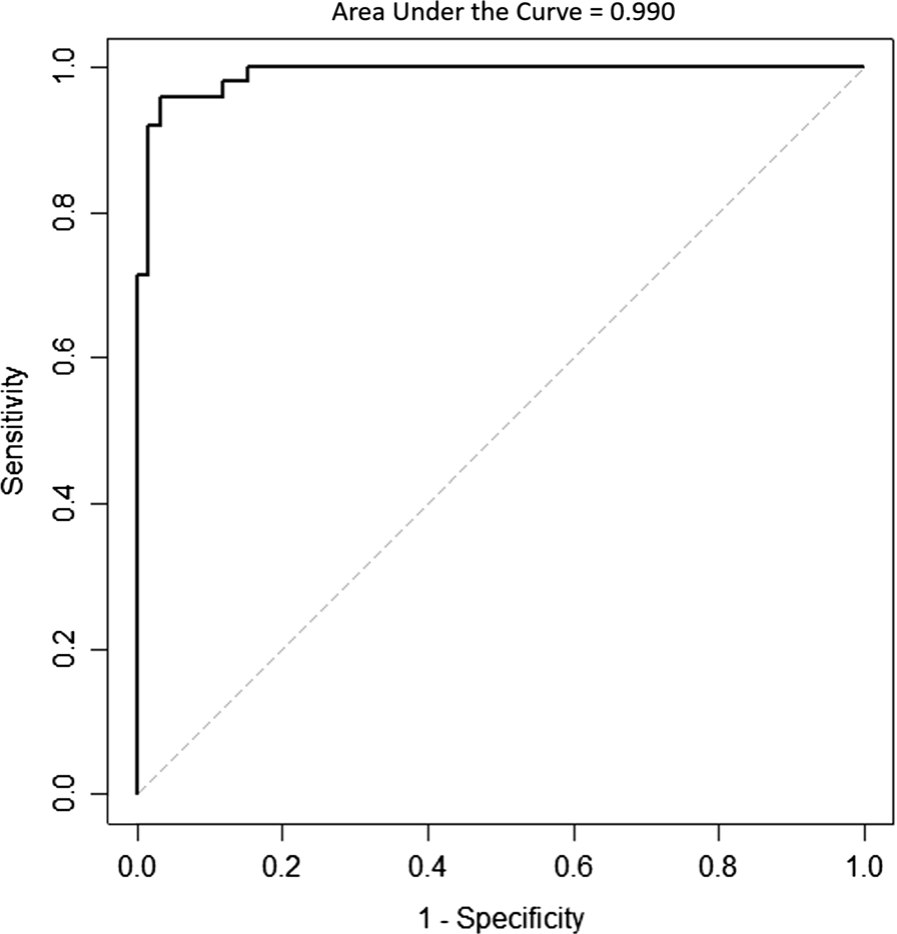
Receiver operating characteristic (ROC) curve in classifying INPH patients and AD patients using lateral ventricle volume/high-convexity subarachnoid space volume ratio

The average lateral ventricle volume/high-convexity subarachnoid space volume ratio of the INPH group was 74.385 ± 80.591 and the average lateral ventricle volume/high-convexity subarachnoid space volume ratio of the AD group was 5.742 ± 3.404 (mean ± standard deviation), a significant difference (*P* < 0.001). The ROC curve showed that a cutoff score of 11.714 on the lateral ventricle volume/high-convexity subarachnoid space volume ratio yielded the highest sensitivity and specificity with regard to differentiating patients with INPH and AD (Fig. 4). Moreover, the area under the ROC curve was 0.990, indicating that this ratio had an excellent discriminant ability.

## Discussion

The findings of our study were as follows: (1) INPH patients had larger lateral ventricles and CSF spaces in the Sylvian fissure region and significantly smaller high-convexity subarachnoid spaces than other groups, and AD patients had larger lateral ventricles and CSF spaces in the Sylvian fissure region than the control group; (2) the INPH group showed a significant negative correlation between lateral ventricle volume and high-convexity subarachnoid space volume, while the AD group showed a significant positive correlation between lateral ventricle volume and volume for CSF spaces of the Sylvian fissure region; and (3) the ratio of lateral ventricle volume to high-convexity subarachnoid space volume was significantly different between INPH and AD groups, and a ROC curve cutoff score of 11.714 yielded high sensitivity and specificity for differentiating INPH and AD patients with an area under the ROC curve of 0.990, indicating excellent discriminatory ability.

Regarding lateral ventricular expansion, our results match previous studies that report INPH patients generally have the largest expansion in that area compared to AD patients and healthy controls. In INPH patients, CSF volume was significantly increased in the ventricles, including lateral, third, and fourth ventricles, in comparison with AD patients [7]. Average ventricular volume, including lateral and third ventricles, in INPH patients was greater than the average ventricular volume in both AD patients and healthy controls [22]. The AD group had greater lateral ventricular enlargement in comparison to controls [23]. That INPH patients also showed the largest CSF spaces of the Sylvian fissure region is an expected result since a previous study has also shown similar results [7]. In addition, INPH patients demonstrated a significant surface expansion primarily in the superior portion of the bilateral lateral ventricles, which are surrounded by the medial frontal lobe and the high convexity of the frontal and parietal regions [24]. And INPH patients had significantly thicker cortices than controls in high convexity areas of the parietal, frontal, and occipital regions [25], which may be due to reactive gliosis [25], known to occur commonly in hydrocephalus [26]. The core process of reactive gliosis is related to cellular hypertrophy, which may be connected to astrocyte proliferation [27], so we cautiously suggest these as possible mechanisms to explain our finding of a significantly smaller high-convexity subarachnoid space in INPH patients.

The negative correlation between lateral ventricle volume and high-convexity subarachnoid space volume in INPH patients, as suggested above, might be explained by cortical thickening around the high convexity area and expansion in the superior portion of the lateral ventricles, which in turn may cause a reduction in the high-convexity subarachnoid space. The positive correlation between lateral ventricle volume and volume for CSF spaces of the Sylvian fissure region may be explained by the type of lateral ventricular dilation in AD patients, hydrocephalus ex vacuo, which is due to atrophy. Medial temporal lobe atrophy is a central characteristic of AD and is one of the first changes seen in the brains of AD patients [28]. And it is well-known that temporal lobe atrophy can also be assessed by evaluating the size of the Sylvian fissure [29]. Atrophic changes to the brain parenchyma in these areas can allow for correspondingly larger CSF spaces in these areas. So the mechanisms explaining these correlations may be quite different between AD and INPH patients, but further studies are needed to investigate these findings and any exact underlying mechanisms that may be involved.

The ratio of lateral ventricle to high-convexity subarachnoid space volumes was markedly different between INPH and AD groups. Possibly combining the biomarkers into a ratio might eliminate confounding factors, such as inter-individual differences, or they might reflect pathological processes more efficiently as a ratio, as was suggested by several studies on CSF amyloid-β and CSF tau [30]. Therefore, it also seemed reasonable to compute the ratio of lateral ventricle volume to high-convexity subarachnoid space volume. We suggest the term “VOSS index” (for the ratio of lateral ventricle to high-convexity subarachnoid space volumes), where “VOSS” is an acronym for “ventricle over subarachnoid space”. Further studies with larger study populations and various statistical tools would be needed to establish this index as a neuroimaging biomarker to distinguish INPH from AD.

The advantages of image-processing approaches applied here are as follows. First, the segmentation methodology including partial volume effect correction that defines extraventricular CSF has allowed us to correctly examine CSF volumes in specific ROIs. Further, each MR imaging was transformed separately into a standardized stereotaxic space, that is, an ICBM 152 template, and we subsequently used the proportional Talairach grid coordinates to define ROIs for the high-convexity area and Sylvian fissure region in this stereotaxic space. A common automated approach to ROI analysis is to spatially normalize each participant’s structural brain image to a template brain image and determine ROIs with an atlas [31]. Second, we used an automated lateral ventricle segmentation method with a graph cuts algorithm combined with a morphological opening and an atlas-based segmentation. This method reduces user bias by introducing atlas-based segmentation results as a starting model for the graph cuts algorithm [18]. Further, the estimation of the partial volume effect of each tissue can help to define an initial model for graph cuts more accurately [18]. The effect of a morphological opening is to take away small features within the image. Therefore, a morphological opening was also introduced to limit false results from a graph cuts approach [18]. In general, a manual definition of ROIs is highly time-consuming and labor-intensive, and it relies on expertise; as a result, it carries the risk of inter-experimenter variability [31]. Further, while some regions of the brain can be reliably defined, other regions do not have clear anatomical landmarks and as a result may be more difficult to differentiate [31]. The combination of the techniques that we describe might thus be useful for detecting morphologically distinctive features of the CSF spaces in INPH patients.

All INPH patients were selected in consecutive order from a prospectively enrolled INPH registry at our hospital. A limitation of this study is that we did not include INPH patients with a negative CSFTT response. We did this to enhance diagnostic certainty of INPH with the CSFTT. INPH patients with a negative CSFTT response were more likely to have other cerebral comorbidities [32]. A second limitation was that we did not determine AD-specific biomarkers in this study. In addition, AD pathology could not be determined in the INPH patients. CSF failure to eliminate possible toxic metabolites can result in amyloid peptide accumulation in AD or INPH patients [33]. CSF stasis in INPH patients may also show AD-like patterns of brain atrophy [34]. However, we also think that there might be justification for utilizing automated volumetric measures of the CSF spaces including lateral ventricles, high-convexity subarachnoid space, and CSF spaces of the Sylvian fissure region in a large study of patients with INPH. A third limitation was that we did not compare the advantages of the image-processing approaches applied here over classical volumetric techniques like the manual tracing method. Nevertheless, a lack of a gold standard for CSF content assessment can prevent obtaining a definitive conclusion regarding the value of different CSF segmentation techniques [35]. Our findings encourage future studies with larger study populations, including other neurodegenerative diseases such as vascular dementia and atypical parkinsonism (e.g., progressive supranuclear palsy), and the ratio of lateral ventricle to high-convexity subarachnoid space volumes to investigate the possibility of utilizing an automated three-dimensional volumetric analysis as an imaging marker to discriminate INPH from its mimics. The diagnostic accuracy of imaging features in INPH has mainly been investigated in comparison to healthy controls or patients with AD [36]. The neurological symptoms in AD often present differently than in INPH [36]. Rather, vascular dementia and atypical parkinsonism such as progressive supranuclear palsy often present with symptoms that can resemble INPH and enlarged cerebral ventricles, and can be challenging differential diagnoses [36].

In conclusion, this study provided an automated three-dimensional volumetric approach to evaluate CSF spaces. Lateral ventricle and high-convexity subarachnoid space volumes were negatively correlated in INPH patients, while lateral ventricle and Sylvian fissure region CSF volumes were positively correlated in AD patients. These associations between CSF volumes suggest that there might be different mechanisms between INPH and AD to explain their respective lateral ventricular dilations. The ratio of lateral ventricle to high-convexity subarachnoid space volumes distinguished INPH from AD with good diagnostic sensitivity and specificity. We propose to refer to this ratio as the VOSS index. These findings may have implications for differential diagnosis between INPH and AD.

## Data Availability

The data that support the findings of this study are available upon request from the corresponding authors (K.K. and U.Y.). The data are not publicly available due to institute policy.

## References

[1] Relkin N, Marmarou A, Klinge P, Bergsneider M, Black PM (2005). Diagnosing idiopathic normal-pressure hydrocephalus. Neurosurgery.

[2] Narita W, Nishio Y, Baba T, Iizuka O, Ishihara T, Matsuda M (2016). High-convexity tightness predicts the shunt response in idiopathic normal pressure hydrocephalus. AJNR Am J Neuroradiol.

[3] O’Keeffe ST, Kazeem H, Philpott RM, Playfer JR, Gosney M, Lye M (1996). Gait disturbance in Alzheimer’s disease: a clinical study. Age Ageing.

[4] Hashimoto M, Ishikawa M, Mori E, Kuwana N (2010). Diagnosis of idiopathic normal pressure hydrocephalus is supported by MRI-based scheme: a prospective cohort study. Cerebrospinal Fluid Res.

[5] Benedetto N, Gambacciani C, Aquila F, Di Carlo DT, Morganti R, Perrini P (2017). A new quantitative method to assess disproportionately enlarged subarachnoid space (DESH) in patients with possible idiopathic normal pressure hydrocephalus: the SILVER index. Clin Neurol Neurosurg.

[6] van den Heuvel DM, ten Dam VH, de Craen AJ, Admiraal-Behloul F, van Es AC, Palm WM (2006). Measuring longitudinal white matter changes: comparison of a visual rating scale with a volumetric measurement. AJNR Am J Neuroradiol.

[7] Kitagaki H, Mori E, Ishii K, Yamaji S, Hirono N, Imamura T (1998). CSF spaces in idiopathic normal pressure hydrocephalus: morphology and volumetry. AJNR Am J Neuroradiol.

[8] Yamashita F, Sasaki M, Takahashi S, Matsuda H, Kudo K, Narumi S (2010). Detection of changes in cerebrospinal fluid space in idiopathic normal pressure hydrocephalus using voxel-based morphometry. Neuroradiology.

[9] Yamashita F, Sasaki M, Saito M, Mori E, Kawaguchi A, Kudo K (2014). Voxel-based morphometry of disproportionate cerebrospinal fluid space distribution for the differential diagnosis of idiopathic normal pressure hydrocephalus. J Neuroimaging.

[10] Ishikawa M, Hashimoto M, Kuwana N, Mori E, Miyake H, Wachi A (2008). Guidelines for management of idiopathic normal pressure hydrocephalus. Neurol Med Chir.

[11] McKhann GM, Knopman DS, Chertkow H, Hyman BT, Jack CR, Kawas CH (2011). The diagnosis of dementia due to Alzheimer’s disease: recommendations from the National Institute on Aging-Alzheimer’s Association workgroups on diagnostic guidelines for Alzheimer’s disease. Alzheimers Dement.

[12] Sled JG, Zijdenbos AP, Evans AC (1998). A nonparametric method for automatic correction of intensity nonuniformity in MRI data. IEEE Trans Med Imaging.

[13] Collins DL, Neelin P, Peters TM, Evans AC (1994). Automatic 3D intersubject registration of MR volumetric data in standardized Talairach space. J Comput Assist Tomogr.

[14] Smith SM (2002). Fast robust automated brain extraction. Human brain mapping.

[15] Zijdenbos AP, Forghani R, Evans AC (2002). Automatic “pipeline” analysis of 3-D MRI data for clinical trials: application to multiple sclerosis. IEEE Trans Med Imaging.

[16] Tohka J, Zijdenbos A, Evans A (2004). Fast and robust parameter estimation for statistical partial volume models in brain MRI. Neuroimage.

[17] Park S, Yoon U (2017). Automated segmentation of the lateral ventricle based on graph cuts algorithm and morphological operations. J Biomed Eng Res.

[18] Kwak K, Yoon U, Lee DK, Kim GH, Seo SW, Na DL (2013). Fully-automated approach to hippocampus segmentation using a graph-cuts algorithm combined with atlas-based segmentation and morphological opening. Magn Reson Imaging.

[19] Desco M, Pascau J, Reig S, Gispert JD, Santos A, Benito C, et al. (eds.) Multimodality image quantification using the Talairach grid. Medical Imaging 2001: Image Processing. International Society for Optics and Photonics. 2001

[20] Andreasen NC, Rajarethinam R, Cizadlo T, Arndt S, Swayze VW, Flashman LA (1996). Automatic atlas-based volume estimation of human brain regions from MR images. J Comput Assist Tomogr.

[21] Dade LA, Gao FQ, Kovacevic N, Roy P, Rockel C, O’Toole CM (2004). Semiautomatic brain region extraction: a method of parcellating brain regions from structural magnetic resonance images. Neuroimage.

[22] Miskin N, Patel H, Franceschi AM, Ades-Aron B, Le A, Damadian BE (2017). Diagnosis of normal-pressure hydrocephalus: use of traditional measures in the era of volumetric MR imaging. Radiology.

[23] Nestor SM, Rupsingh R, Borrie M, Smith M, Accomazzi V, Wells JL (2008). Ventricular enlargement as a possible measure of Alzheimer’s disease progression validated using the Alzheimer’s disease neuroimaging initiative database. Brain.

[24] Kang K, Kwak K, Yoon U, Lee JM (2018). Lateral ventricle enlargement and cortical thinning in idiopathic normal-pressure hydrocephalus patients. Sci Rep.

[25] Kang K, Han J, Lee SW, Jeong SY, Lim YH, Lee JM (2020). Abnormal cortical thickening and thinning in idiopathic normal-pressure hydrocephalus. Sci Rep.

[26] Miller JM, McAllister JP (2007). Reduction of astrogliosis and microgliosis by cerebrospinal fluid shunting in experimental hydrocephalus. Cerebrospinal Fluid Res.

[27] Sofroniew MV (2005). Reactive astrocytes in neural repair and protection. Neuroscientist.

[28] de Leeuw FE, Korf E, Barkhof F, Scheltens P (2006). White matter lesions are associated with progression of medial temporal lobe atrophy in Alzheimer disease. Stroke.

[29] Davis PC, Mirra SS, Alazraki N (1994). The brain in older persons with and without dementia: findings on MR, PET, and SPECT images. AJR Am J Roentgenol.

[30] De Vos A, Struyfs H, Jacobs D, Fransen E, Klewansky T, De Roeck E (2016). The cerebrospinal fluid neurogranin/BACE1 ratio is a potential correlate of cognitive decline in Alzheimer’s disease. J Alzheimers Dis.

[31] Garrison KA, Rogalsky C, Sheng T, Liu B, Damasio H, Winstein CJ (2015). Functional MRI preprocessing in lesioned brains: manual versus automated region of interest analysis. Front Neurol.

[32] Kang K, Ko PW, Jin M, Suk K, Lee HW (2014). Idiopathic normal-pressure hydrocephalus, cerebrospinal fluid biomarkers, and the cerebrospinal fluid tap test. J Clin Neurosci.

[33] Silverberg GD, Mayo M, Saul T, Rubenstein E, McGuire D (2003). Alzheimer’s disease, normal-pressure hydrocephalus, and senescent changes in CSF circulatory physiology: a hypothesis. Lancet Neurol.

[34] Hilal S, Xin X, Ang SL, Tan CS, Venketasubramanian N, Niessen WJ (2015). Risk factors and consequences of cortical thickness in an Asian population. Medicine.

[35] Via E, Cardoner N, Pujol J, Martinez-Zalacain I, Hernandez-Ribas R, Urretavizacaya M (2012). Cerebrospinal fluid space alterations in melancholic depression. PLoS ONE.

[36] Fällmar D, Andersson O, Kilander L, Löwenmark M, Nyholm D, Virhammar J (2021). Imaging features associated with idiopathic normal pressure hydrocephalus have high specificity even when comparing with vascular dementia and atypical parkinsonism. Fluids Barriers CNS.

